# Prospective assessment of plate-haptic rotationally asymmetric multifocal toric intraocular lens with near addition of + 1.5 diopters

**DOI:** 10.1186/s12886-020-01731-3

**Published:** 2020-11-18

**Authors:** Tetsuro Oshika, Kazuno Negishi, Toru Noda, Hiroyuki Arai, Mikio Inamura, Yasushi Inoue, Teruyuki Miyoshi, Yoshifumi Fujita, Kazunori Miyata, Yumi Hasegawa

**Affiliations:** 1 Department of Ophthalmology, Faculty of Medicine, University of Tsukuba, 1-1-1 Tennoudai, Tsukuba, Ibaraki, 305-8575 Japan; 2 Department of Ophthalmology, Keio University School of Medicine, Tokyo, Japan; 3 Department of Ophthalmology, National Hospital Organization, Tokyo Medical Center, Tokyo, Japan; 4 Queen’s Eye Clinic, Yokohama, Kanagawa Japan; 5 Inamura Eye Clinic, Yokohama, Kanagawa Japan; 6 Inoue Eye Clinic, Tamano, Okayma Japan; 7 Miyoshi Eye Clinic, Fukuyama, Hiroshima Japan; 8 Fujita Eye Clinic, Tokushima, Tokushima Japan; 9 Miyata Eye Hospital, Miyakonojo, Miyazaki Japan

**Keywords:** Intraocular lens, Multifocal lens, Toric lens, Rotationally asymmetric lens, Refractive segmented lens

## Abstract

**Background:**

To prospectively evaluate surgical results following implantation of rotationally asymmetric, plate-haptic, refractive segmented multifocal toric intraocular lenses (IOLs) with near addition of + 1.5 diopters (D) (Lentis Comfort LS-313 MF15T, Oculentis GmbH).

**Methods:**

In 59 eyes of 41 patients, ocular examinations were conducted before and 1 day, 1 week, 1, 3, and 6 months after surgery. Uncorrected (UDVA) and corrected (CDVA) distance visual acuity, uncorrected (UIVA) and distance-corrected (DCIVA) intermediate visual acuity at 70 cm, and uncorrected (UNVA) and distance-corrected (DCNVA) near visual acuity at 30 cm were tested. A defocus curve was drawn, and the degree of disturbing photic phenomena were questioned.

**Results:**

The IOL showed excellent rotational stability; the average absolute rotation was 1.66 ± 1.17 degrees from 1 day 1 to 6 months postoperatively, and 98.1 and 100% of eyes yielded rotation of less than 5 and 10 degrees, respectively. Postoperative distance and intermediate visual acuity were highly satisfactory; UDVA, CDVA, UIVA, and DCIVA were about 20/20, 20/16, 20/25, 20/25, respectively. Near visual acuity was suboptimal; UNVA and DCNVA were at approximately 20/60. The defocus curve analysis showed that 20/25 and 20/40 uncorrected visual acuity was attained at as close as 60 and 40 cm, respectively. Contrast sensitivity was within a normal range, and subjective photic phenomena were minimum.

**Conclusions:**

The refractive segmented, rotationally asymmetric multifocal toric IOLs with + 1.5 D near addition showed superb rotational stability and highly satisfactory distance and intermediate vision. Contrast sensitivity was high and incidence of photic symptoms was very low.

**Trial registration:**

This study was registered at JAPIC Clinical Trials Information, ID: JapicCTI-183,877, https://www.clinicaltrials.jp/cti-user/trial/Search.jsp (February 5, 2018).

## Background

The rotationally asymmetric, refractive segmented intraocular lens (IOL) with + 1.5 diopter (D) addition (Lentis Comfort LS-313 MF15, Oculentis GmbH, Berlin, Germany) is a relatively new generation of multifocal IOL that is designed to boost intermediate performance while suppressing disturbing photic phenomena [1–6]. This IOL does not compromise contrast sensitivity of patients, and the frequency and degree of glare and halo are similar to those of monofocal IOLs [6], leading to widespread acceptance within a monofocal IOL segment in several markets. Lentis Comfort LS-313 MF15T is the toric version of this IOL and expected to further enhance patients’ visual performance. The unique configuration of this type of lens, plate-haptic design, necessitates sufficient investigation on the rotational stability after surgery, but no such study has been available until now. The current multicenter, 6-month prospective study was conducted to evaluate surgical outcomes of rotationally asymmetrical, refractive multifocal IOL with near addition of + 1.5 D.

## Methods

### Patient recruitment

This multicenter, prospective study was a 6-month phase III clinical trial to assess Lentis Comfort toric LS-313 MF15T and to file for approval from the Ministry of Health, Labor and Welfare of Japan. Patients with age-related cataract having corneal cylinder between 0.75 D and 2.5 D were selected from clinic population. Eyes with previous history of ocular surgery were not included. Eyes were also precluded from the subjects if they had any ocular pathologies that can influence surgical results.

The study protocol was approved by the institutional review board at all surgical centers (Keio University, National Hospital Organization Tokyo Medical Center, Queen’s Eye Clinic, Inamura Eye Clinic, Inoue Eye Clinic, Miyoshi Eye Clinic, Fujita Eye Clinic, and Miyata Eye Hospital). Preoperatively, each patient gave informed consent in a written form. This study adhered to the Declaration of Helsinki tenets and good clinical practice guide for medical devices in Japan (Pharmaceuticals and Medical Devices Agency: PMDA clinical trial identifier: TC2). This study was registered at JAPIC Clinical Trials Information, ID: JapicCTI-183,877, https://www.clinicaltrials.jp/cti-user/trial/Search.jsp (February 5, 2018).

### Intraocular lenses and surgery

The IOL used in the current study was a foldable hydrophilic IOL of a hydrophobic acrylic material. It is a plate-haptic, rotationally asymmetric, refractive multifocal toric IOL, consisting of a zone for distance vision and a sector-shaped zone for intermediate vision. The addition power is + 1.5 D at the lens plane. The + 1.5 D addition at the IOL plane is calculated to be + 1.06 D addition at the corneal plane. The optic is 6.0 mm in diameter and the overall length of the plate-haptic is 11.0 mm [7–9]. There was three toric models; T1 has 1.5 D toricity at the IOL plane and 1.04 D toricity at the corneal plane, T2 has 2.25 D and 1.56 D, and T3 has 3.0D and 2.08 D. The toric axis is fixed on the horizontal meridian of the IOL. All eyes were targeted emmetropia.

Eight surgeons from 8 surgical sites conducted surgeries, using a standard technique of phacoemulsification with a self-sealing 2.3- or 2.4-mm incision. After removal of nucleus and cortex through anterior capsulorhexis of approximately 5.0 mm in diameter, the IOL was implanted into the capsular bag using a specific injector recommended by the manufacturer (ACCUJECT UNIFIT WJ-60 M II, Santen Pharmaceutical Co., Ltd. Osaka, Japan, or Viscojet-BIO 2.2 injector. Medicel AG, Wolfhalden, Altenrhein).

### Examinations

The ocular examinations were carried out preoperatively and 1 day, 1 week, 1, 3, and 6 months postoperatively. Preoperative measurements included uncorrected (UDVA) and corrected (CDVA) distance visual acuity, uncorrected (UIVA) and distance-corrected (DCIVA) intermediate visual acuity measured at 70 cm, uncorrected (UNVA) and distance-corrected (DCNVA) near visual acuity measured at 30 cm, intraocular pressure, manifest refraction, keratometry, slitlamp anterior segment examination, optical biometry, and retina evaluation under pupil dilation.

After surgery, visual acuity at distance, intermediate, and near were measured at every postoperative visit. At 6 months postoperatively, a defocus curve was created for 15 different levels of defocus from + 2.0 to − 5.0 D in steps of 0.5 D. The contrast sensitivity was assessed using a CSV-1000 chart (Vector Vision, Greenville, OH) at 3, 6, 12, and 18 cycles per degree. The background illumination for the translucent chart was provided by the fluorescent luminance source that was automatically calibrated to 85 cd/m^2^. Before and 6 months after surgery, endothelial cell density was recorded using a noncontact automated specular microscopy (EM-3000, TOMEY, Aichi, Japan, or SP-3000P, Topcon, Tokyo, Japan).

The IOL axis rotation was evaluated according to the methods described by Schartmüller et al. [10] At 1 day, 1 week, 1, 3, and 6 months postoperatively, high-resolution, slit-lamp digital retroillumination photographs were taken with a dilated pupil, and non-moving episcleral vessels or Axenfeld loops were marked with a photo editing software. The same principle was applied for measurements during the follow-up visits by marking the identical landmarks. Two axes were drawn, one between specific location of the lens to assign the IOL axis and one between two typical landmarks. The angle between these two lines was measured to determine the degree of IOL rotation from day 1, independent from head movement or ocular cyclotorsion. The absolute value of rotation was calculated and averaged. One experienced physician (YH) conducted all image analyses.

The subjective severity of photic phenomena was assessed. The intensity of glare and halo was graded from none, mild, moderate, to severe. The degree of difficulty in night vision categorized from none, mild to moderate, to severe. The overall satisfaction with surgical outcomes were asked as very high, high, medium, and low. The any episode of any intraoperative and postoperative adverse effects was recorded throughout the study period.

### Sample size calculation

Considering that 61% of eyes with Lentis-313 MF15 attained UDVA of 20/20 or better [2], in order that 38.4% (the approval request form for production and distribution of medical devices, toric IOLs) [11, 12] reach UDVA of 20/20 or better, the number of cases required was calculated to be 42 eyes (α = 0.05, power 80%). In addition, UIVA of 20/20, 20/25, and 20/32 were obtained in 58, 66.67, and 100% of eyes with this IOL, respectively [2], and approximately 80% of eyes with a representative multifocal toric IOL in the market achieved UIVA of 20/40 (Acrysof IQ ReSTOR toric multifocal IOL summary of safety and effectiveness data). In order that 95% of eyes clear UIVA of 20/40, the number of necessary cases was calculated to be 48 eyes (α = 0.05, power 80%). A 10% dropout rate was anticipated, leading to the target sample size of 53 eyes.

### Statistical analysis

Numerical data are expressed as mean ± standard deviation. Statistical comparisons between two paired measurements were performed using the paired t-test. The measurement results of three groups (T1, T2, and T3) were compared using the Kruskal-Wallis test. Statistical analysis was performed using SPSS Statistics for Windows software (version 26, IBM Corp., Armonk, NY, USA). In all cases, the level of significance was a *p*-value less than 0.05.

## Results

The patient’s demographics are summarized in Table 1. There was no case of mature cataract, and all eyes presented mild to moderate cataract. The enrolment of patients started February 2018, and all follow-up examinations finished December 2018. All patients completed the 6-month pre-determined examination schedule.

**Table 1:** Demographics

Number	59 eyes of 41 patients
Age (years)	69.9 ± 9.3 (43–87)
Male:Female	11:30
Axial length (mm)	23.73 ± 0.77 (22.15–25.12)
Radius of anterior corneal curvature (mm)	7.67 ± 0.25 (7.20–8.39)
Preoperative corneal astigmatism (D)	1.66 ± 0.77 (0.75–4.0)
T1:T2:T3	30:20:9
Power of intraocular lens (D)	19.2 ± 2.0 (16.0–23.5)

Mean ± SD (range), D: diopters

**Fig. 1:**
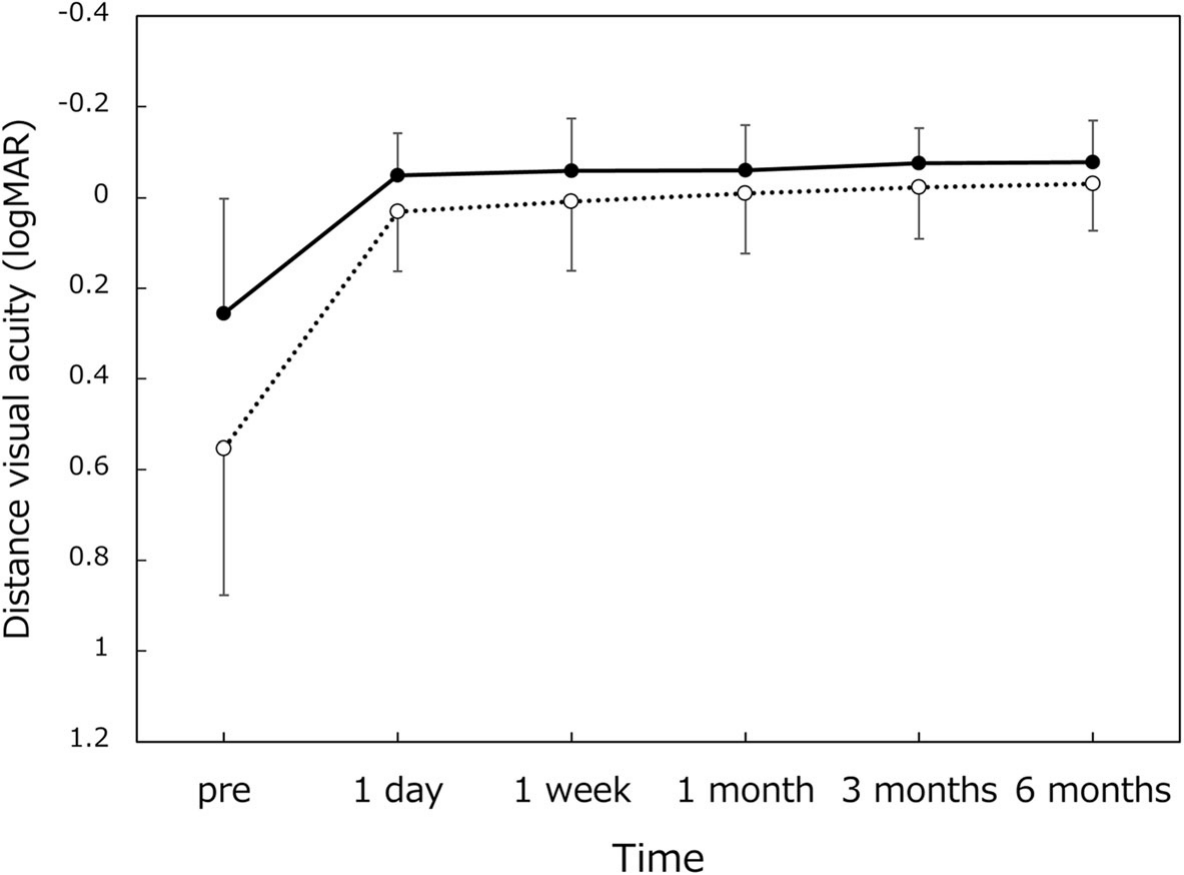
Changes in uncorrected (dotted line) and corrected (solid line) distance visual acuity over time. logMAR = logarithm of minimum angle of resolution, mean ± standard deviation

**Fig. 2:**
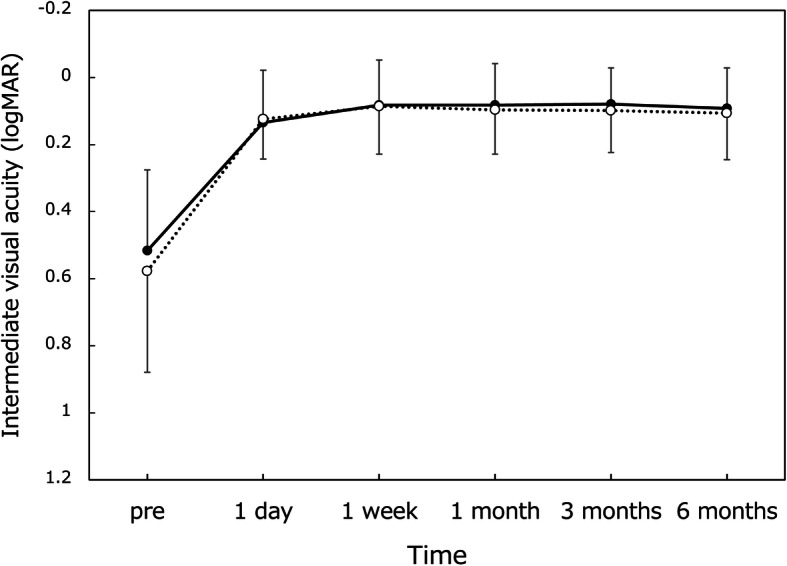
Changes in uncorrected (dotted line) and distance-corrected (solid line) intermediate visual acuity over time. logMAR = logarithm of minimum angle of resolution, mean ± standard deviation

**Fig. 3:**
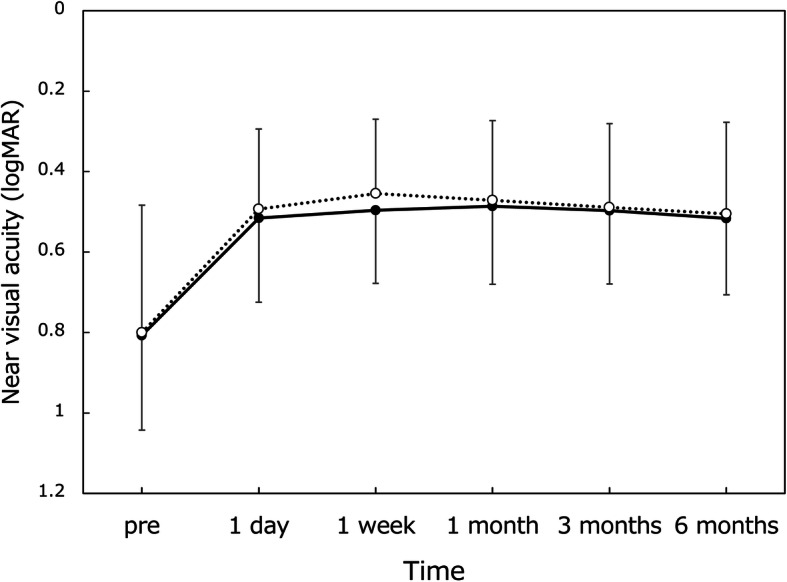
Changes in uncorrected (dotted line) and distance-corrected (solid line) near visual acuity over time. logMAR = logarithm of minimum angle of resolution, mean ± standard deviation

Good distance visual acuity (Fig. 1) was obtained throughout 6 months, with UDVA and CDVA of approximately 20/20 and 20/16, respectively. For intermediate visual acuity (Fig. 2), UIVA and DCIVA of around 20/25 were obtained after surgery. On the other hand, near visual acuity was lower compared with distance and intermediate visual acuity (Fig. 3). UNVA and DCNVA were at the level of around 20/60.

**Fig. 4:**
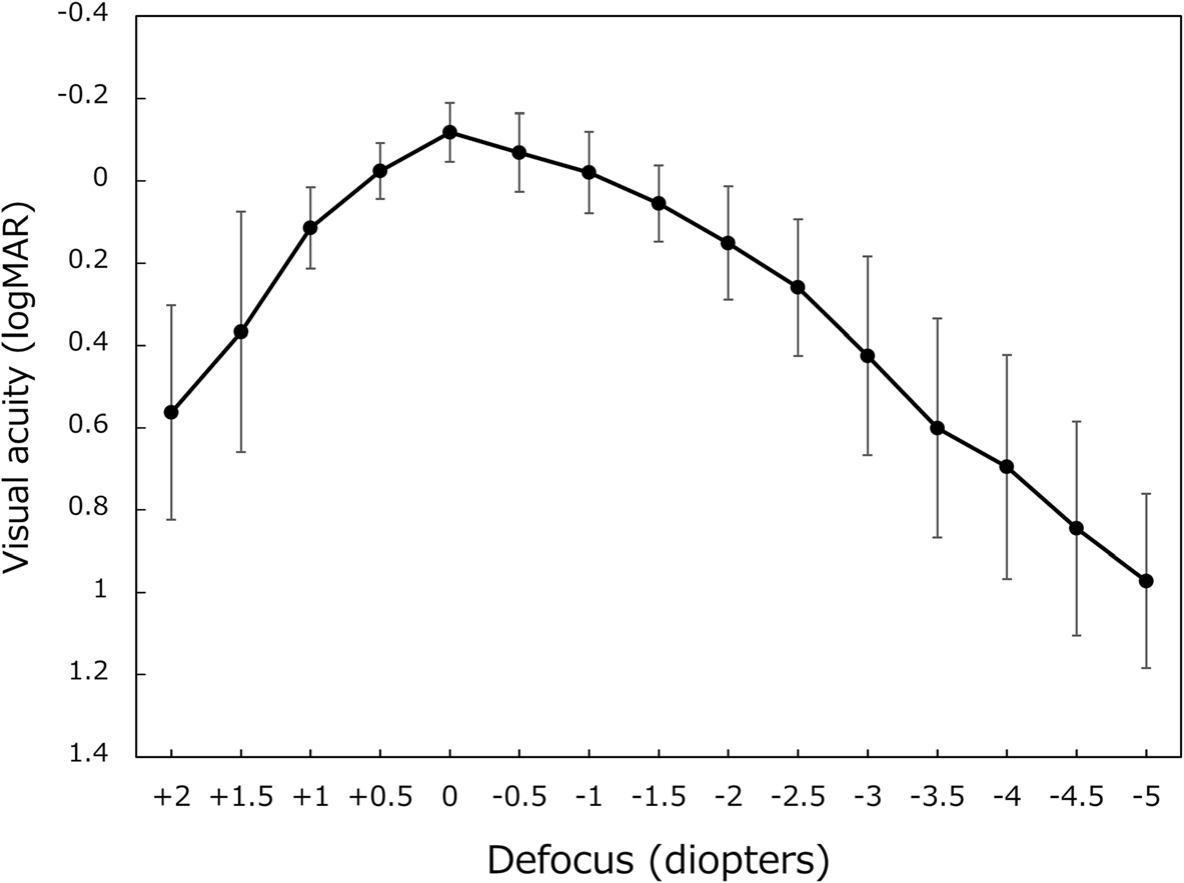
Defocus curve from + 2.0 to − 5.0 diopters. logMAR = logarithm of minimum angle of resolution, mean ± standard deviation

The defocus curve created at 6 months postoperatively (Fig. 4) indicated that 20/25, 20/30, and 20/40 vision was reached in the defocus range of + 0.5 to − 1.7 D, + 1.0 to − 2.0 D, and + 1.3 to − 2.6 D, respectively. These results show that postoperative uncorrected visual acuity of 20/25 and 20/40 were attained at as close as 60 and 40 cm in distance, respectively.

**Fig. 5:**
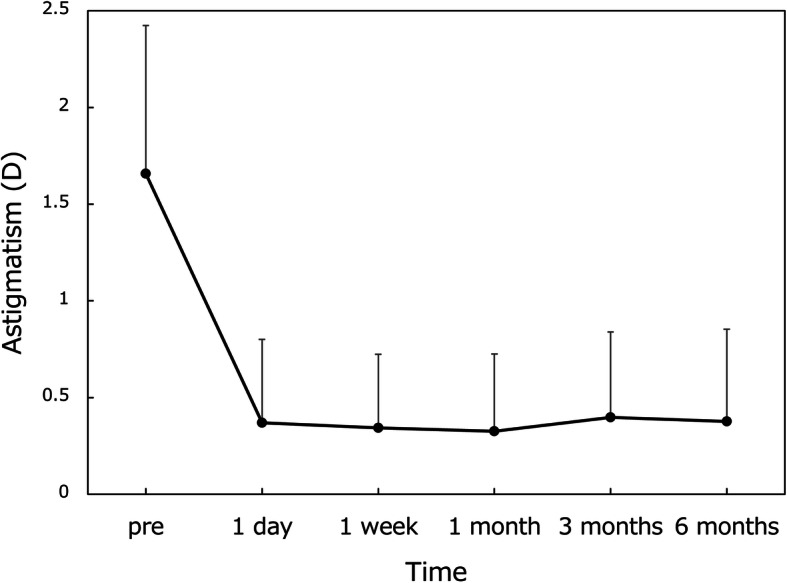
Preoperative corneal astigmatism and postoperative manifest refractive astigmatism. Mean ± standard deviation

Preoperative astigmatism of 1.66 ± 0.77 D (corneal) before surgery was significantly reduced to manifest refractive astigmatism of 0.32 ~ 0.40 D after surgery (Fig. 5). Residual astigmatism at 6 months postoperatively was 0.38 ± 0.50 D, 0.41 ± 0.52 D, and 0.28 ± 0.31 D in eyes with toric models T1, T2, and T3, respectively (*p* = 0.878, Kruskal-Wallis test).

**Fig. 6:**
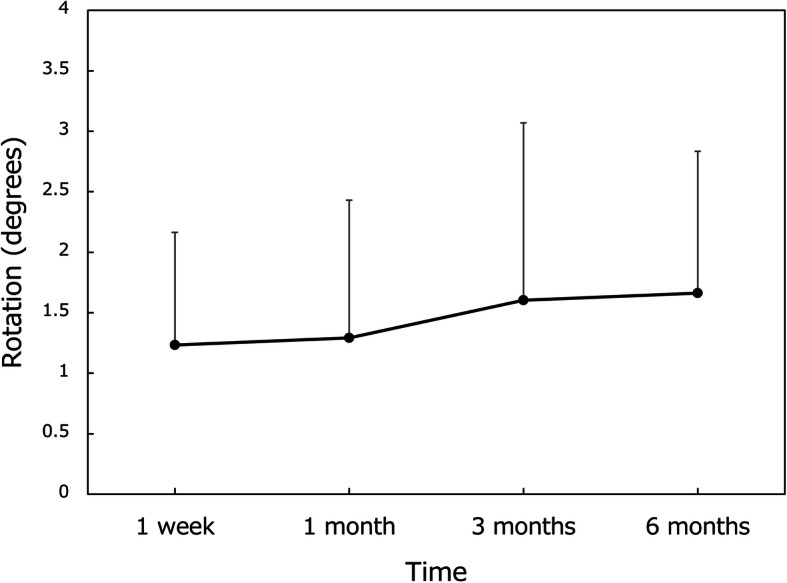
Rotation of toric IOL axis from 1 day after surgery. Mean ± standard deviation

The time course of changes in IOL rotation (absolute value) from 1 day after surgery is shown in Fig. 6. The average amount of absolute rotation at 6 months postoperatively was 1.66 ± 1.17 degrees, with 98.1% (53/54) of eyes showing rotation of less than 5 degrees and 100% (54/54) within 10 degrees’ rotation, where 5 eyes could not be analyzed due to poor image quality of the photographs. IOL rotation from 1 day to 6 months postoperatively was 1.73 ± 1.24 degrees, 1.46 ± 1.10 degrees, and 1.92 ± 1.18 degrees in eyes with toric models T1, T2, and T3, respectively (*p* = 0.549, Kruskal-Wallis test). IOL repositioning surgery was performed in one eye at 22 days postoperatively for misalignment of 19 degrees, which was corrected to 3.6 degrees thereafter.

**Fig. 7:**
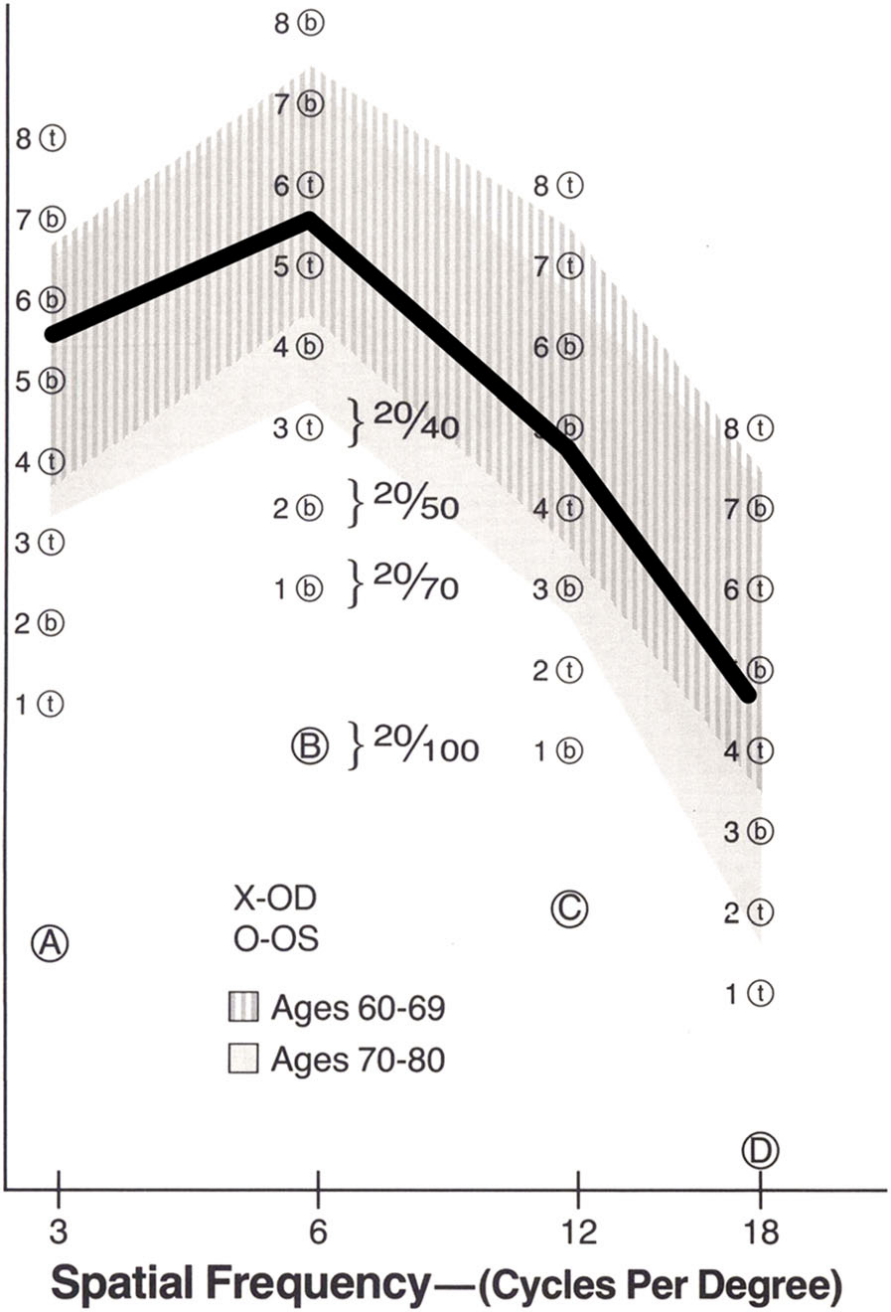
Contrast sensitivity function at 6 months postoperatively. Results are within the normal range

Contrast sensitivity function investigated at 6 months postoperatively is plotted in Fig. 7. The results were within the range of age-considered normal controls.

Incidence and intensity of subjective disturbing photic symptoms are shown for glare (Table 2), halo (Table 3), and difficulty in night vision (Table 4). The level of disturbing photic symptoms was low, and none of the patients complained severe photic symptoms. As for overall satisfaction, majority if patients rated very high or high (Table 5). There were no gender differences in these subjective scores.

**Table 2:** Incidence and severity of glare

	1 day ~ 1 week	1 month	3 months	6 months
None	32 (54.2%)	53 (89.9%)	54 (91.5%)	56 (96.6%)
Mild	8 (13.6%)	5 (8.5%)	5 (8.5%)	2 (3.4%)
Medium	16 (27.1%)	1 (1.7%)	0	0
Severe	3 (5.1%)	0	0	0

**Table 3:** Incidence and severity of halo

	1 day ~ 1 week	1 month	3 months	6 months
None	36 (61.0%)	55 (93.2%)	55 (93.2%)	56 (96.6%)
Mild	9 (15.3%)	3 (5.1%)	4 (6.8%)	1 (1.7%)
Medium	12 (20.3%)	1 (1.7%)	0	1 (1.7%)
Severe	2 (3.4%)	0	0	0

**Table 4:** Incidence and severity of difficulty in night vision

	1 day ~ 1 week	1 month	3 months	6 months
None	14 (23.7%)	52 (88.1%)	54 (91.5%)	54 (93.1%)
Mild to moderate	36 (61.0%)	7 (11.9%)	5 (8.5%)	4 (6.9%)
Severe	9 (15.3%)	0	0	0

**Table 5:** Overall satisfaction

	1 day ~ 1 week	1 month	3 months	6 months
Very high	0	42 (71.2%)	43 (72.9%)	46 (79.3%)
High	1 (1.7%)	17 (28.8%)	16 (27.1%)	12 (20.7%)
Medium	43 (72.9%)	0	0	0
Low	15 (25.4%)	0	0	0

The corneal endothelial cell density was 2666 ± 283 /mm^2^ preoperatively and 2615 ± 298 /mm^2^ at 6 months postoperatively. There was no intraoperative complication. One eye (1.7%) was treated for posterior capsule opacification with YAG laser posterior capsulotomy at 3 months postoperatively, and CDVA recovered from 20/25 to 20/16. There was no case of other postoperative complications, such as IOL decentration and tilt.

## Discussion

We found that the rotation of this plate-haptic, multifocal toric IOL was minimal throughout the 6-month study period. The average amount of absolute rotation at 6 months postoperatively was 1.66 ± 1.17 degrees, with 98.1% of eyes showing rotation of less than 5 degrees and 100% within 10 degrees’ rotation. The pre-existing astigmatism of 1.66 ± 0.77 D was significantly reduced by surgery to less than 0.40 D on average. These results indicate that the current plate-haptic, rotationally asymmetric multifocal toric IOL is highly effective in the correction of pre-existing astigmatism at the time of cataract surgery. Garzón et al. [13] evaluated the monofocal version of similar plate-haptic toric IOL (Lentis LT) at 1 months, and reported that mean misalignment was 3.71 ± 5.94 degrees. They also demonstrated that 64.6% of eyes presented IOL misalignment of less than 5 degrees, and more than 10 degrees’ misalignment was observed in 14.28%. Venter et al. [14] assessed refractive outcomes and rotational stability after implantation of multifocal (+ 3.0 D add) toric IOL with a surface-embedded near section (Lentis Mplus LS-312), and reported that the mean difference between the planned axis of implantation and the actual axis orientation at 3 months postoperatively was 2.53 ± 2.27 degrees. All IOLs were within ±10 degrees of the intended axis, and 89.9% were within ±5 degrees.

Throughout the 6-month follow-up period, the distance visual acuity remained highly satisfactory; UDVA and CDVA were approximately 20/20 and 20/16, respectively. The intermediate vision was also maintained at a high level; both UIVA and DCIVA hovered at around 20/25. Near visual acuity, on the other hand, stayed at a lower level, with UNVA and DCNVA of about 20/60. With this level of near visual acuity, one can read large size prints, but not small prints, necessitating reading aids. It was reported that asymmetric multifocal IOLs with near addition of + 1.5 D yielded superb distance vision (UDVA of 0.00 logMAR and CDVA of − 0.08 logMAR) in conjunction with good intermediate vision (UIVA at 80 cm of 0.01 logMAR), but near vision was rather limited (UNVA at 40 cm of 0.41 logMAR) [2]. Another study indicated that Lentis Comfort LS-313 MF15 gave excellent distance and intermediate visual acuity (UDVA of − 0.01 logMAR and UIVA at 70 cm of 0.05 logMAR), while near visual acuity was at a lower level (UNVA at 30 cm of 0.54 logMAR) [4]. The findings of our study are in good agreement with these previous reports.

The defocus curve at 6 months postoperatively (Fig 4) indicated a gradually declining pattern from distance, intermediate, to near, unlike the 2-peak curve given by the conventional distance-near bifocal IOLs with a larger addtion [1]. Other studies also presented a defocus curve with a similar single peak with Lentis Comfort LS-313 MF15 IOL [2–5]. The defocus curve in our study indicates that uncorrected visual acuity of 20/25 or better can be obtained at as close as 60 cm (− 1.7 D), and 20/40 or better at 40 cm (− 2.6 D) in distance. These results are better than those reported for non-toric Lentis MF15 [6], where 20/25 and 20/40 were achieved at as close as 67 cm (− 1.5 D) and 20/40 and 45 cm (− 2.2 D), respectively, indicating that addition of toricity can enhance patients’ vision.

Contrast sensitivity function measured at 6 months postoperatively was within the normal range of age-matched controls. It has been shown that contrast sensitivity of this IOL is similar to that of standard monofocal IOLs [4–6]. Previous studies reported that the level of contrast sensitivity did not differ between extended-range-of-vision IOLs and monofocal IOLs, but these two IOLs exhibited significantly better level of contrast sensitivity than conventional multifocal IOLs with + 2.0 D or + 3.0 D addition [15]. The contrast sensitivity test evaluates persons’ ability to perceive low contrast images, while the standard vision test assesses how well persons can identify black-on-white high contrast letters. With the use of low contrast optotypes, the contrast sensitivity test can assess slight changes in vision that cannot be revealed by the regular vision test. For the investigation of quality of vision in patients implanted with premium IOLs, the contrast sensitivity test is often utilized.

Incidence and degree of disturbing photic symptoms were evaluated, such as glare, halo, and difficulty in night vision. During the first week after surgery, there were a few cases of medium to severe photic phenomena, but those symptoms were quickly mitigated thereafter. After 1 week postoperatively, no case complained of severe photic phenomena, and patient’s satisfaction reached a very high level. This process appears to reflect the neuroadaptation of patients to multifocal IOLs [16–18]. It has been known that difficulties associated with photic phenomena after implantation of conventional multifocal and trifocal IOLs decreased significantly over time [19, 20].

It was reported that not many patients complained of disturbing photic symptoms after implantation of the rotationally asymmetric, segmented, refractive multifocal IOL with addition of + 1.5 D [1, 3, 4, 6]. Yoo et al. [1] compared visual function in eyes with multifocal Lentis comfort LS-313 MF15 (+ 1.5D add) and Lentis M plus LS-313 MF30 (+ 3.0 D add), and exhibited that the former IOL was associated with significantly fewer halo and glare than the latter type. The low-add concept of this IOL yields an elongated focal zone without multiple foci as indicated by the defocus curve, leading to minimum apparent out-of-focus images that may generate halo phenomena. This can explain the low incidence of disturbing photic symptoms observed with this IOL.

This study has several limitations. First, there was no control group. It should be more preferable to design a head-to-head comparative study with other monofocal or other multifocal lenses with or without toricity. The present study plan was agreed on with and approved by the Ministry of Health, Labor and Welfare of Japan, as an open-label study to compare with past IOL studies. Second, postoperative examination started 1 day after surgery in our study. In previous studies [10, 21], toric IOL axis alignment was measured at the end of surgery as well as 1 h after surgery to elucidate the time course of changes in toric IOL axis orientation in detail. In the current multicenter study involving 8 surgical sites, however, it was not possible to design and conduct such demanding schedules.

## Conclusion

Our prospective, multicenter study found that rotationally asymmetrical, sector-shaped multifocal toric IOL with near addition of + 1.5 D provides highly satisfactory distance and intermediate vison, with good contrast sensitivity as well as minimum subjective photic symptoms. On the other hand, near visual acuity remained suboptimal for small print reading. Rotational stability was excellent throughout the 6-month study period.

## Data Availability

The datasets generated during and/or analysed during the current phase III clinical trial were used to file for approval from the Ministry of Health, Labor and Welfare of Japan. The data are not publicly available but are available from Santen Pharmaceutical on reasonable request.

## Abbreviations

IOL: Intraocular lens
D: Diopter
UDVA: Uncorrected distance visual acuity
CDVA: Corrected distance visual acuity
UIVA: Uncorrected intermediate visual acuity
DCIVA: Distance-corrected intermediate visual acuity
UNVA: Uncorrected near visual acuity
DCNVA: Distance-corrected near visual acuity
